# Estimation of Total Phenols, Flavanols and Extractability of Phenolic Compounds in Grape Seeds Using Vibrational Spectroscopy and Chemometric Tools

**DOI:** 10.3390/s18082426

**Published:** 2018-07-26

**Authors:** Berta Baca-Bocanegra, Julio Nogales-Bueno, Francisco José Heredia, José Miguel Hernández-Hierro

**Affiliations:** Food Colour and Quality Laboratory, Área de Nutrición y Bromatología, Facultad de Farmacia, Universidad de Sevilla, 41012 Sevilla, Spain; bbaca1@us.es (B.B.-B.); heredia@us.es (F.J.H.); jmhhierro@usal.es (J.M.H.-H.)

**Keywords:** phenolic compounds, extractability, total phenols, flavanols, grape seeds, near infrared, vibrational spectroscopy, chemometrics

## Abstract

Near infrared hyperspectral data were collected for 200 Syrah and Tempranillo grape seed samples. Next, a sample selection was carried out and the phenolic content of these samples was determined. Then, quantitative (modified partial least square regressions) and qualitative (K-means and lineal discriminant analyses) chemometric tools were applied to obtain the best models for predicting the reference parameters. Quantitative models developed for the prediction of total phenolic and flavanolic contents have been successfully developed with standard errors of prediction (SEP) in external validation similar to those previously reported. For these parameters, SEPs were respectively, 11.23 mg g^−1^ of grape seed, expressed as gallic acid equivalents and 4.85 mg g^−1^ of grape seed, expressed as catechin equivalents. The application of these models to the whole sample set (selected and non-selected samples) has allowed knowing the distributions of total phenolic and flavanolic contents in this set. Moreover, a discriminant function has been calculated and applied to know the phenolic extractability level of the samples. On average, this discrimination function has allowed a 76.92% of samples correctly classified according their extractability level. In this way, the bases for the control of grape seeds phenolic state from their near infrared spectra have been stablished.

## 1. Introduction

There is a high variability of phenolic compounds in grapes (*Vitis vinifera* L.). These compounds can be found in the whole berry (skin, pulp and seeds) and in the fermentation stage, they become part of the wine [1]. Depending on varieties, grape seeds contains up to half of the berry phenolic compounds, and they represent between 4% and 6% of the seed weight [2]. In grape seed, the most important phenolic compounds are flavanols (or flavan-3-ols). They can be found as monomers ((+)-catechin, (−)-epichatechin or epichatechin 3-galate) oligomers or polymers [3]. Moreover, phenolic acids (benzoic or hidroxycinnamic acids) are also found in grape seeds [4]. These phenolic compounds play an important role in the sensory characteristics of wine. They are typically linked to the flavor (acidity, bitterness and astringency) and color (via copigmentation phenomena) of red wines [5].

Therefore, knowing the amount of phenols that are transferred to wine from grape seeds (i.e., extractable total phenolic content or extractable phenolic content) is an essential issue in the wine industry, since the quality of wine depends largely on this aspect. Extractable phenolic content largely depends on the total amount of phenolic compounds that grape seeds have (i.e., total phenolic content). In consequence, it is necessary to define the extractability as the ratio between the extractable phenolic content and the total phenolic content. Phenolic extractability allows comparing samples with different total phenolic content and sorting samples according to their phenolic extractability.

There are a number of methods that allow obtaining the extractable or total content of the more important phenolic families. For example, in grape seed samples, the Folin-Ciocalteu [6] and 4-dimethyl-aminocinnamaldehyde (DMACA) [7] methods can be applied in order to obtain the extractable or total content of total phenols and flavanols, respectively (being total phenols the totality of phenolic compounds present in grape seeds, i.e., phenolic acids, flavanols, flavonols, etc.). These kind of traditional methods for the control of parameters of interest in grapes are being replaced by non-destructive and green chemistry methods. Among them, a high number of near infrared hyperspectral methods have been developed in the last decade in order to screen several parameters of interest in grapes [8]. In some studies, hyperspectral imaging is applied to predict total or extractable phenolic content in grapes or grape seeds [9,10], to control phenolic or technological maturity [11] or to control the composition of oenological by-products [12].

In this study, hyperspectral imaging has been applied to control the extractable phenolic content, the total phenolic content and the extractability of phenolic compounds. In particular, flavanols and total phenols have been studied. Near infrared spectra have been acquired for two hundred samples of Syrah and Tempranillo grape seeds and a sample selection procedure has been carried out. Next, reference parameters, extractable and total contents and phenolic extractability of total phenols and flavanols, have been chemically evaluated for selected samples. Then a number of chemometric approaches have been interrogated (PCA, MPLS, K-means cluster analysis and LDA) in order to obtain the best methods for predicting the reference parameters. Finally, the developed methods have been applied to all samples with the exception of spectral outliers and the obtained distributions of the reference parameters have been evaluated in the samples.

## 2. Materials and Methods

### 2.1. Samples

Grape seeds from two hundred Syrah and Tempranillo grapes (*Vitis vinifera* L.) were used in this study. The procedures carried out for grape collection and grape seed extraction from the whole grapes are described in detail elsewhere in [13]. Briefly, two hundred Syrah and Tempranillo red grape samples were collected from two vineyards located in the Condado de Huelva Designation of Origin D.O. (Andalusia, Spain) on two different dates (7 and 11 August 2014). In order to achieve representative samples sets, single grapes were collected from the top, middle and bottom of the cluster and from the sunlight and shade side. The samples were refrigerated and immediately transported to the laboratory.

### 2.2. Acquisition of Hyperspectral Data

Hyperspectral images of grape seeds belonging to an individual grape were jointly acquired. Hyperspectral data collection is described in Rodríguez-Pulido, et al. [10]. Briefly, hyperspectral imaging device (Infaimon S.L., Barcelona, Spain) comprised a Xenics^®^ XEVA-USB InGaAs camera (320 × 256 pixels; Xenics Infrared Solutions, Inc., Leuven, Belgium), a spectrograph (Specim ImSpector N17E Enhanced; Spectral Imaging Ltd., Oulu, Finland) covering the spectral range between 900 and 1700 nm (spectral resolution of 3.25 nm). Samples were placed 40 cm below the camera’s scanning window and two 70 W tungsten iodine halogen lamps (Prilux, Barcelona, Spain) were used as lighting sources at 45° from the vertical.

**Figure 1:**
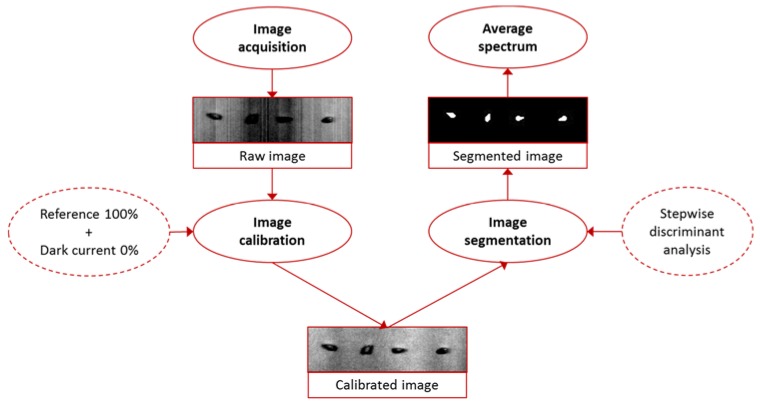
Description of the procedure carried out for each sample from the raw hyperspectral image acquisition until obtaining the average spectrum.

Raw hyperspectral images were corrected from the dark current effect, then, the regions of interest (i.e., pixels belonging to grape seeds) were selected. The selection of the regions of interest was carried out by means of a forward stepwise discriminant analysis. For that, a number of spectra belonging to seeds and background (a homogeneous surface composed of polyethylene) were manually collected and they were used for the creation of the algorithm of segmentation. The algorithm saved all the masks of segmentation and they were visually supervised for ensuring the suitability of the proposed method. Then, the average reflectance spectra were obtained for each grape seed sample. Next, spectra were transformed to relative absorbances and the spectral region comprised between 950 and 1650 nm was saved and a spectral matrix (200 samples × 215 wavelengths) was formed. Figure 1 describes the whole procedure carried out for each sample from the spectra acquisition until the obtaining the average spectrum.

### 2.3. Sample Selection

A sample selection procedure was carried out to reduce the dimension of the spectral matrix without losing of significant spectral information and to decrease the number of seed samples to be chemically analyzed. Sample selection was carried out following a modification of the method of Nogales-Bueno, et al. [14] as it is described in detail in [13]. In brief, a principal component analysis (PCA) was applied to the near infrared spectral matrix and, as result, 66 groups of samples spectrally different were identified. Next, calibration and validation sets were created by allocating one sample from every group respectively. Therefore, the calibration set consisted of 66 samples, while the validation set was composed of only 26 samples because there were two or more samples in only 26 groups. Finally, the weight of these 92 seed samples was measured and they were conserved at −20 °C until chemical analyses were carried out.

### 2.4. Phenolic Characterization of Grape Seeds: Extractable Content, Total Content and Extractability of Total Phenols and Flavanols

Extractable total phenolic content (EPC), extractable flavanolic content (EFC), total phenolic content (TPC), total flavanolic content (TFC), extractability of total phenols (ETP) and extractability of flavanols (EF) were measured for samples selected in Section 2.3 and these variables were used as reference parameters in the subsequent chemometric approaches.

Extractable contents were determined by the analysis of the supernatants of grape seeds extractions in model wine (12.5% (*v*/*v*) ethanol, 4 g L^−1^ tartaric acid, adjusted at pH 3.6 with NaOH 0.5 M). A ratio of 25 mL of model wine per each gram of seed was kept constant for all samples. These macerations were carried out at room temperature in a dry place during 72 h without any external agitation. Supernatants were used for the determination of the extractable contents. Next, they were freeze-dried, grounded and macerated in methanol:water 75:25 (*v*/*v*), sonicated during 15 min (JP Selecta, Barcelona, Spain) and centrifuged (830× *g*, 15 min). This solution was added in a constant ratio of 10 mL g^−1^ for all samples. These extractions were repeated twice in order to achieve an exhaustive extraction of phenolic compounds. The methanolic extracts were combined and finally made up to a final volume of 50 mL with methanol. These supernatants were analyzed and the results combined with those obtained from the model macerations and total contents of total phenols and flavanols were obtained.

EPC and TPC were determined using the Folin-Ciocalteu method [6]. Two hundred microliters of exhaustive or model wine supernatants were mixed with 1.5 mL of sodium carbonate (20% *w*/*v*), 500 µL of Folin reagent and made up to 10 mL with ultrapure water.

In order to measure EFC and TFC, a modification of Vivas et al. [7] method was carried out. Ten or twenty microliters of exhaustive or model wine supernatants were mixed with 190 μL or 180 μL of methanol respectively and 1 mL of DMACA (4-dimethylaminocinnamaldehyde) reagent.

Both Folin-Ciocalteau and DMACA analyses were performed on an Agilent 8453 UV–Visible spectrophotometer (Palo Alto, CA, USA), equipped with diode array detection (DAD), measuring absorbance at 765 and 640 nm respectively. The extract volumes were appropriately modified for samples which needed it. For quantification, Folin-Ciocalteau results were expressed as mg of gallic acid equivalents per gram of grape seed, whereas DMACA results were expressed as mg of catechin equivalents per gram of grape seed.

Finally, ETP and EF of each sample were evaluated as follows:

(1) $$ETP=\frac{EPC}{TPC}\times 100;\;EF=\frac{EFC}{TFC}\times 100$$

### 2.5. Data Analysis

#### 2.5.1. Quantitative Calibrations

Raw spectral data of samples allocated in the calibration set were used to develop a quantitative calibration for each reference parameter. The corresponding reference parameters were allocated to each sample and different spectral pretreatments were tested. A number of spectral pretreatments, such as standard normal variate (SNV), multiplicative scatter correction (MSC) or detrending, were applied to spectral samples allocated in the calibration set in order to remove the scattering effects [15,16]. Moreover, the effect of differentiation and variations in spectral ranges were tested in the development of the NIRS calibrations. Afterwards, a modified partial least squares (MPLS) regression was performed for each reference parameter. In MPLS regression, calibration samples are split in different subsets. In this way, a cross-validation is performed, the possibility of overfitting is reduced, the number of PLS factors are set and chemical outliers are removed [17]. Detection of chemical outliers was performed following a *T* ≥ 2.5 criterion and these samples were not taken into account in the MPLS regression due to their high residual predicted value. Finally, the standard error of cross-validation was obtained by the combination of the validation errors in a single figure.

A number of statistics were used to evaluate the performance of the obtained calibration models. The applicability range of the models is defined by the maximum and minimum estimations and, jointly with the standard deviation (SD), allows knowing what data can be used for an external validation. The standard error of calibration (SEC) and standard error of cross-validation (SECV) are estimates of the prediction capability of the equation. It is considered that SECV statistic is similar to the average standard error of prediction (SEP) from 10 randomly chosen prediction sets. The multiple correlation coefficient (RSQ) measures how well the calibration fits the data. Finally, standard error of prediction (SEP) compares the real with the predicted values obtained for the reference parameter. It is obtained SEP in internal validation if this comparison is made for samples that do belong to the calibration set, else it is obtained SEP in external validation. In this study, external validations were carried out.

Quantitative models, sample selection, PCA and data pretreatments were carried out in Win ISI^®^ (v1.50) software (Infrasoft International, LLC, Port Matilda, PA, USA).

#### 2.5.2. K-Means Cluster Analysis

K-means cluster analysis was performed using Statistica v.8.0 software (StatSoft Inc., Tulsa, OK, USA). Samples were classified according to their extractability of phenolic compounds (i.e., ETP and EF). Initial between-cluster distances were maximized by choosing the appropriate initial cluster centers. Then, two groups of samples were stablished according to their phenolic extractability levels: Low and high extractability groups.

#### 2.5.3. Supervised Pattern Recognition Analysis

Linear discriminant analysis (LDA) was applied in the present study as supervised pattern recognition method. This method was carried out using the prior probabilities of classification and the size of each group was taken into account. Samples correctly classified were considered in order to estimate the prediction ability of the method. For that, leave-one-out cross-validation and external validation were applied. The variables used were the scores of the 8 first PCs performed on the near infrared hyperspectral data. All variables were used in the analysis. SPSS 22.0 (SPSS, Inc., Chicago, IL, USA) was used for the LDA implementation.

## 3. Results and Discussion

### 3.1. Near Infrared Hyperspectral Data

**Figure 2:**
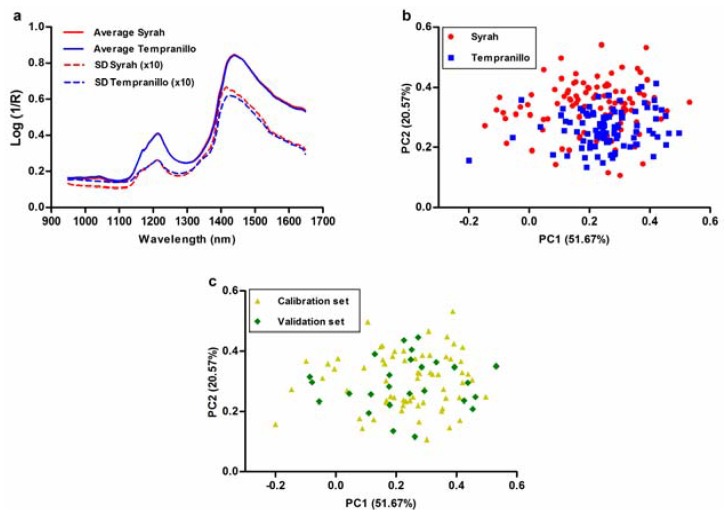
Description of near infrared spectra. (**a**) Near infrared average raw spectra and standard deviations (10 times amplified) for Syrah and Tempranillo samples. (**b**) Scores of the grape samples in the space defined by the first and second PCs. (**c**) Scores of the calibration and validation samples in the space defined by PC1 and PC2.

In Figure 2, near infrared spectra are described. Figure 2a shows the average raw spectra and the standard deviations (amplified 10 times) for Syrah and Tempranillo samples. Average raw spectra are quite similar to each other in the whole spectral range. Figure 2b shows the scores of the grape samples in the space defined by the first and second PCs which described 51.67% (PC1) and 20.57% (PC2) of the spectral variability in the data. There is not a separation between Syrah and Tempranillo samples. However, Syrah samples are more scattered than Tempranillo ones, being Tempranillo samples mainly in the right and down side of the space defined by PC1 and PC2. In this space are also shown the scores of the validation and calibration samples (Figure 2c). Although sample selection was carried out taking into account the first 8 PCs, the comparison between Figure 2b,c shows that almost all the spectral variability of samples are included in the validation and calibration sets.

### 3.2. Chemical Analysis

The main statistical descriptors for extractable content, total content and extractability of total phenols and flavanols of the samples allocated in the validation and calibration sets were obtained (Table 1). These values are comparatively similar than those described in bibliography [10,18]. Taking into account these statistical descriptors, it can be inferred that in calibration set chemical variability is bigger than in validation one. These results are surely linked with the spectral relationship between both sample sets.

### 3.3. Quantitative Calibrations

Samples allocated into the calibration set were used to perform MPLS regressions. In these quantitative calibrations, the 66 seed spectra were used as independent (X) variables. Reference parameters (EPC, EFC, TPC, TFC, ETP and EF) previously determined for grape seed samples were used as dependent (Y) variables. The statistical parameters of the final calibration equations are shown in Table 2 where N is the number of samples used to obtain the calibration equation after eliminating samples for chemical reasons (T criterion). The mathematical treatment applied (i.e., the best of the different treatment interrogated), the range of application, and standard deviations are also shown for each reference parameter.

External validations were carried out for each selected model. For, TPC, TFC, ETP and EF all samples presented reference values within the applicability of the obtained models. However, in the case of EPC and EFC, one Syrah sample presented reference values outside of the applicability range of the obtained models. Therefore, this sample was removed from the validation set in these validation procedures. In Table 2 were also included the standard errors of prediction (SEP) in external validation obtained in the validation of each reference parameter. 

For TPC and TFC, similar errors have been reported by other authors, taking into account the applicability range, for total or extractable contents of these compounds using near infrared spectroscopy [10,12,18,19,20]. For the interpretation of these errors it is necessary to take into account the standard error of the reference methods. These errors, for the determinations of total phenols and flavanols, are around 10% [6,7,21,22]. Therefore, these variables can be considered appropriated to be used as reference parameters. In consequence, MPLS regressions developed from grape seed NIR spectra present a good potential for a fast and reasonably inexpensive screening of total contents of total phenols and flavanols (TPC and TFC respectively) in these samples.

**Figure 3:**
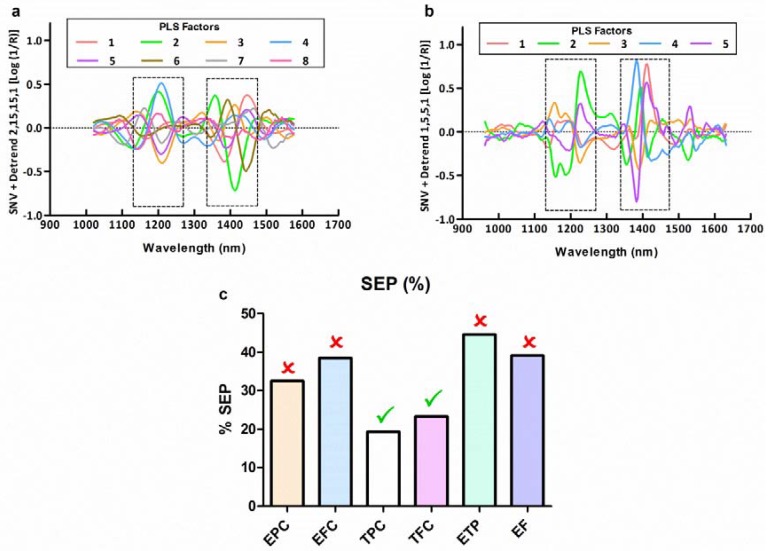
(**a**) Loading plots of the MPLS model for total phenolic content (TPC). (**b**) Loading plots of the MPLS model for total flavanolic content (TFC). (**c**) Standard errors of prediction obtained in the external validation procedure for all MPLS models carried out expressed as percentages.

The loading plots of the MPLS models for TPC and TFC are shown in Figure 3a,b, respectively. The loadings show important features in the spectral regions around 1200 and 1400 nm. These regions are usually ascribed to combination bands of the –OH functional group and symmetric and antisymmetric stretching. Moreover, second and third overtones of C−H aromatic bond are also assigned to this band. These features can be attributed to the chemical structure of the analyzed compounds [23,24,25]. 

These spectral regions have been identified as important regions in other similar studies. Zhang et al. [19] predict total phenols in grape seeds and they identify the regions about 1200 and 1450 nm as the regions with a high importance in the prediction. In the case of the prediction of flavanols, Ferrer-Gallego et al. [18] and Rodríguez-Pulido et al. [10] also declare the importance of the spectral regions about 1100–1300 and 1400 nm. In the case of EPC, EFC, ETP and EF, the standard errors of prediction obtained in the external validation procedure were too high (Figure 3c), not being possible the correct prediction of these parameters by the use of MPLS regressions. Due to the high importance of these parameters, other approaches were carried out for ETP and EF in order to link the phenolic extractability in grape seeds to their spectral features in the near infrared region.

### 3.4. Qualitative Analysis for the Control of the Extractability of Phenolic Compounds

Grape seed samples were sorted according to their extractability of phenolic compounds (ETP and EF). For this purpose, a k-means analysis was carried out. Taking into account these two variables, k-means cluster analysis sorted grape seed samples in two different groups. Then, these groups were named as low and high extractability levels. Samples of calibration and validation sets were both sorted. By the application of the k-means method, they were obtained the number of seed samples classified as samples with low or high extractability and, then, the mean and standard deviation for ETP and EF were obtained for these samples (Table 3).

Afterwards, an LDA was carried out in order to discriminate samples according their extractability level (high or low). LDA was carried out using the scores of the 8 first PCs obtained from near infrared hyperspectral data, which had previously been used for the sample selection (expressed as PC1 to PC8 for simplicity). Results of this LDA are shown in Table 4. The results of the classification of grape seed samples according to their extractability level of phenolic compounds reveal a good percentage of correctly classified samples. The model classifies correctly the 83.3% of the samples in leave-one-out cross-validation and the 76.9% of the samples in external validation. Table 4 also shows the lineal discriminant function. If the scores of the 8 first PCs obtained from near infrared hyperspectral data are known for other samples, this discrimination function can be applied for the classification of these grape seed samples according to their extractability. Respectively, the standardized canonical coefficients (*β*) for the scores of the first 8 PCs are: 0.678, −0.628, 0.547, 0.295, −0.187, −0.520, −0.042 and 0.596. Therefore, the variables with the greatest influence on the discrimination are PC1 and PC2 scores.

If the loadings of the PCA are analyzed, important features are found in the spectral regions around 1200 and 1400 nm (data not shown). As mentioned above, similar results were found in the loading plots of the MPLS models. Therefore, the importance of these spectral regions is confirmed.

### 3.5. Application of the Developed Tools in the Control of Grape Seed Phenols

#### 3.5.1. Total Phenolic and Flavanolic Contents

**Figure 4:**
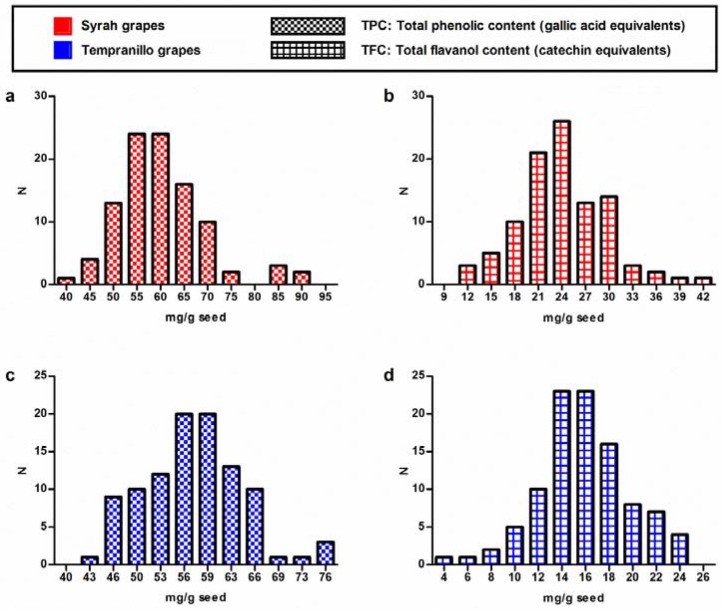
Distributions of Syrah and Tempranillo grape seeds in different total phenolic content (**a**,**c**) and total flavanolic content (**b**,**d**).

By applying the quantitative calibration models developed in previous sections, total phenolic and total flavanolic contents were predicted for the whole set of collected grape seeds samples with the exception of the spectral outlier. Models described in Table 2 were applied to a total of 199 samples (99 Syrah and 100 Tempranillo samples) for the prediction of TPC and TFC. Figure 4 shows the distributions of Syrah and Tempranillo grape seeds in different total phenolic content (a and c) and total flavanolic content (b and d). It can be appreciated that, in all cases, the two parameters describe a Gaussian bell-shaped distribution. This confirms the heterogeneity found within the same ripeness stage for the above-said parameters. It is noteworthy that similar results were found in a previous study for extractable polyphenols in Syrah and Tempranillo grape skin [14].

Basic statistical descriptors of predicted values (mean and standard deviation) indicate higher values of TPC and TFC for Syrah samples than for Tempranillo. For TPC (expressed as gallic acid equivalents) these descriptors were for Syrah samples, respectively, 60.14 mg g^−1^ and 9.59 mg g^−1^ and for Tempranillo samples, 57.31 mg g^−1^ and 6.97 mg g^−1^. Whereas for TFC (expressed as catechin equivalents), these statistics were for Syrah samples, respectively: 24.21 mg g^−1^ and 5.51 mg g^−1^ and for Tempranillo samples 15.98 mg g^−1^ and 3.84 mg g^−1^.

**Figure 5:**
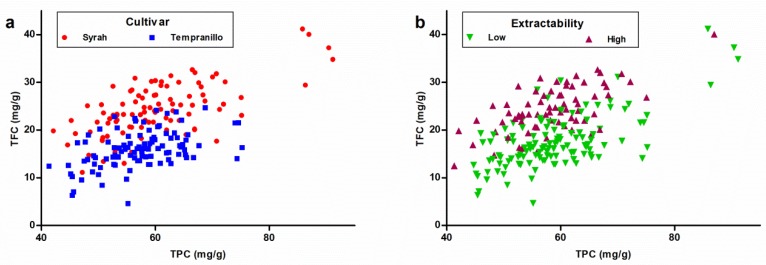
Representation of grape seed samples according their predicted total phenolic content (TPC) and total flavanolic content (TFC). Samples are codified as (**a**) Syrah or Tempranillo samples or (**b**) samples with low or high phenolic extractabilities.

In Figure 5a, samples are plotted according to their TPC and TFC values. It can be observed that, in most cases, Syrah samples have a higher amount of TFC than Tempranillo samples. Regarding to TPC, differences are lower than in the previous case, although five Syrah samples show really high total contents.

#### 3.5.2. Phenolic Extractability Levels

In order to determine the extractability level for the whole grape seed set (spectral outlier was not taken into account), the discriminant function previously obtained (Table 4) was applied. For these samples, scores of the first 8 PCs were introduced in the discriminant function and samples were classified according to their phenolic extractability level as samples with low or high extractability level.

As result, 127 grape seeds were classified as samples with low extractability level and the remaining 72 seeds samples as samples with high extractability level. Among high extractability samples, 69 were Syrah samples whereas only 3 where Tempranillo. In consequence, low extractability group was composed of 30 Syrah and 97 Tempranillo samples. Those results indicate higher extractability of phenolic compounds in Syrah than in Tempranillo seeds. This higher extractability can be attributed to the physiological differences among both varieties. For example, Tempranillo grapes generally present a more mature state than other varieties on similar dates. In this way, Tempranillo grapes present more mature seeds than Syrah grapes and it is well-known that phenolic extractability in grape seeds decreases during ripening due to, among others, changes in the cell wall polysaccharide structure and lignification [10,13,26].

In Figure 5b, samples of different extractability levels are plotted separately according to their TPC and TFC values. It is noteworthy that samples with the highest total contents (TPC and TFC) are not always samples with a high extractability level. Although these samples have high total contents, they do not necessarily release phenolic compound easier than samples with a lower total amount of these compounds. Nevertheless, in a large number of cases and especially for TFC, samples with high content are samples of high extractability.

## 4. Conclusions

Quantitative models carried out in this work, from near infrared hyperspectral images, provide good results for the screening of total phenolic and flavanolic contents in grape seeds in a fast and reasonably inexpensive way. These models have errors which are comparatively similar to the errors previously reported for these parameters in bibliography. Moreover, spectral region with high importance in the prediction of these parameters have been identified and the heterogeneity of total polyphenols within the same ripeness stage has been observed.

Qualitative models have also been carried out for the identification of grape seed samples with low or high phenolic extractability levels. The model classifies correctly the 83.3% of the samples in leave-one-out cross-validation and the 76.9% of the samples in external validation. By the application of the developed model, higher extractabilities of phenolic compounds have been found in Syrah than in Tempranillo seeds.

In this preliminary study, a number of simplifications have been adopted to obtain the feasibility of using the hyperspectral imaging in the control of phenolic extractability in grape seeds. These simplifications are intended to simulate a post-fermentative process. In future studies, it would be interesting to recalculate these chemometric models for pre-fermentative or fermentative processes. For example, ethanol or temperature variations, regular agitation, changes in pH, production of enzymes or the formation of new polyphenols during the fermentation may be taken into account.

## Figures and Tables

**Table 1:** Main statistical descriptors for reference parameters in calibration and validation sets.

Set	Reference Parameter	Maximum	Mean	Minimum	SD ^1^
Calibration	EPC ^2^	79.92	17.14	0.67	15.39
	EFC ^3^	56.42	11.07	0.34	11.22
	TPC ^4^	99.97	59.40	32.90	13.51
	TFC ^5^	66.92	21.66	6.63	10.49
	ETP ^6^	80.59	28.09	0.99	20.15
	EF ^7^	89.16	42.60	2.20	25.76
Validation	EPC ^2^	41.89	14.91	2.24	9.49
	EFC ^3^	33.12	10.17	0.99	8.54
	TPC ^4^	88.14	56.62	27.50	13.37
	TFC ^5^	39.42	20.65	11.03	8.21
	ETP ^6^	82.91	29.06	4.04	22.01
	EF ^7^	93.04	44.23	7.80	25.33

^1^ SD: Standard deviation; ^2^ EPC: extractable total phenolic content (mg g^−1^ of grape seed, expressed as gallic acid equivalents); ^3^ EFC: extractable flavanolic content (mg g^−1^ of grape seed, expressed as catechin equivalents); ^4^ TPC: total phenolic content (mg g^−1^ of grape seed, expressed as gallic acid equivalents); ^5^ TFC: total flavanolic content (mg g^−1^ of grape seed, expressed as catechin equivalents); ^6^ ETP: extractability of total phenols (expressed as percentages); ^7^ EF: extractability of flavanols (expressed as percentages).

**Table 2:** Main statistical descriptors for the MPLS models developed in the NIR zone close to 950–1650 nm.

Spectral Pretreatment	Reference Parameters	T Outliers	PLS Factors	N ^1^	Est. Min	SD ^2^	Est. Max	SEC ^3^	RSQ ^4^	SECV ^5^	SEP ^6^
None 2,10,10,1	EPC ^7^	5	5	61	0.00	9.17	41.80	5.13	0.69	6.45	6.79
None 2,10,10,1	EFC ^8^	6	5	60	0.00	7.59	31.80	3.50	0.79	4.21	6.12
SNV + Detrend 2,15,15,1	TPC ^9^	2	8	64	23.22	11.64	93.08	7.38	0.60	8.41	11.23
SNV + Detrend 1,5,5,1	TFC ^10^	3	5	63	0.00	7.19	41.62	2.17	0.91	3.62	4.85
None 2,15,15,1	ETP ^11^	2	6	64	0.00	19.62	86.36	9.74	0.75	11.83	19.26
None 2,15,15,1	EF ^12^	0	6	66	0.00	25.76	119.87	13.50	0.73	16.67	23.47

^1^ N: number of samples (calibration set); ^2^ SD: standard deviation; ^3^ SEC: standard error of calibration; ^4^ RSQ: coefficient of determination (calibration set); ^5^ SECV: standard error of cross-validation (7 cross-validation groups); ^6^ SEP: standard error of prediction (external validation); ^7^ EPC: extractable total phenolic content (mg g^−1^ of grape seed, expressed as gallic acid equivalents); ^8^ EFC: extractable flavanolic content (mg g^−1^ of grape seed, expressed as catechin equivalents); ^9^ TPC: total phenolic content (mg g^−1^ of grape seed, expressed as gallic acid equivalents); ^10^ TFC: total flavanolic content (mg g^−1^ of grape seed, expressed as catechin equivalents); ^11^ ETP: extractability of total phenols (expressed as percentages); ^12^ EF: extractability of flavanols (expressed as percentages).

**Table 3:** Extractability levels of total phenols and flavanols for grape seed samples allocated in calibration and validation sets. Means and standard deviations are shown.

Set	Samples	N ^1^	ETP ^2^		EF ^3^	
			Mean	SD	Mean	SD
Calibration	All	66	28.09	20.15	42.60	25.76
	Low	36	12.93	8.08	22.39	13.83
	High	30	46.30	14.24	66.84	11.87
Validation	All	26	29.06	22.01	44.23	25.33
	Low	14	13.76	6.20	24.73	10.71
	High	12	46.91	20.24	66.97	16.58

^1^ N: number of samples; ^2^ ETP: extractability of total phenols (expressed as percentages); ^3^ EF: extractability of flavanols (expressed as percentages).

**Table 4:** Samples correctly classified by the LDA in the leave-one-out cross-validation and in the external validation. The obtained lineal discriminant function is also shown.

Samples	Leave-One-Out Cross-Validation		External Validation	
	Samples Correctly Classified	% of Samples Correctly Classified	Samples Correctly Classified	% of Samples Correctly Classified
Low	30/36	83.33	12/14	85.7
High	25/30	83.33	8/12	66.67
All	55/66	83.33	20/26	76.92
Discriminant function	*D* = −17.316 + 4.735PC1 − 6.993PC2 + 9.199PC3 + 8.307PC4 − 6.770PC5 − 21.565PC6 − 1.608PC7 + 24.292PC8			

## References

[1] Waterhouse A.L. (2002). Wine Phenolics.

[2] Ribéreau-Gayon P., Dubourdieu D., Doneche B., Lonvaud A., Glories Y., Maujean A., Branco J.M. (2006). Handbook of Enology, The Microbiology of Wine and Vinifications.

[3] Ristic R., Iland P.G. (2005). Relationships between seed and berry development of Vitis Vinifera L. cv Shiraz: Developmental changes in seed morphology and phenolic composition. Aust. J. Grape Wine Res..

[4] Jara-Palacios M.J., Gordillo B., González-Miret M.L., Hernanz D., Escudero-Gilete M.L., Heredia F.J. (2014). Comparative Study of the Enological Potential of Different Winemaking Byproducts: Implications in the Antioxidant Activity and Color Expression of Red Wine Anthocyanins in a Model Solution. J. Agric. Food Chem..

[5] Ribéreau-Gayon P., Glories Y., Maujean A., Dubourdieu D. (2006). Handbook of Enology, The Chemistry of Wine: Stabilization and Treatments.

[6] Singleton V.L., Rossi J.A. (1965). Colorimetry of Total Phenolics with Phosphomolybdic-Phosphotungstic Acid Reagents. Am. J. Enol. Vitic..

[7] Vivas N., Glories Y., Lagune L., Saucier C., Augustin M. (1994). Estimation du degré de polymérisation des procyanidines du raisin et du vin par la méthode au p-dimethylaminocinnamaldéhyde. J. Int. Sci. Vigne Vin..

[8] Nogales-Bueno J., Rodríguez-Pulido F.J., Baca-Bocanegra B., González-Miret M.L., Heredia F.J., Hernández-Hierro J.M., Gorawala P., Mandhatri S. (2016). Hyperspectral Imaging—A Novel Green Chemistry Technology for the Oenological and Viticultural Sectors. Agricultural Research Updates.

[9] Chen S., Zhang F., Ning J., Liu X., Zhang Z., Yang S. (2015). Predicting the anthocyanin content of wine grapes by NIR hyperspectral imaging. Food Chem..

[10] Rodríguez-Pulido F.J., Hernández-Hierro J.M., Nogales-Bueno J., Gordillo B., González-Miret M.L., Heredia F.J. (2014). A novel method for evaluating flavanols in grape seeds by near infrared hyperspectral imaging. Talanta.

[11] Nogales-Bueno J., Hernández-Hierro J.M., Rodríguez-Pulido F.J., Heredia F.J. (2014). Determination of technological maturity of grapes and total phenolic compounds of grape skins in red and white cultivars during ripening by near infrared hyperspectral image: A preliminary approach. Food Chem..

[12] Jara-Palacios M.J., Rodríguez-Pulido F.J., Hernanz-Vila M.D., Escudero-Gilete M.L., Heredia F.J. (2016). Determination of phenolic substances of seeds, skins and stems from white grape marc by near-infrared hyperspectral imaging. Aust. J. Grape Wine Res..

[13] Nogales-Bueno J., Baca-Bocanegra B., Rooney A., Hernández-Hierro J.M., Byrne H.J., Heredia F.J. (2017). Study of phenolic extractability in grape seeds by means of ATR-FTIR and Raman spectroscopy. Food Chem..

[14] Nogales-Bueno J., Baca-Bocanegra B., Rodríguez-Pulido F.J., Heredia F.J., Hernández-Hierro J.M. (2015). Use of near infrared hyperspectral tools for the screening of extractable polyphenols in red grape skins. Food Chem..

[15] Geladi P., MacDougall D., Martens H. (1985). Linearization and Scatter-Correction for Near-Infrared Reflectance Spectra of Meat. Appl. Spectrosc..

[16] Dhanoa M.S., Lister S.J., Barnes R.J. (1995). On the Scales Associated with Near-Infrared Reflectance Difference Spectra. Appl. Spectrosc..

[17] Shenk J.S., Westerhaus M.O. (1995). Routine Operation,Calibration, Development and Network System Management Manual.

[18] Ferrer-Gallego R., Hernández-Hierro J.M., Rivas-Gonzalo J.C., Escribano-Bailón M.T. (2010). Feasibility study on the use of near infrared spectroscopy to determine flavanols in grape seeds. Talanta.

[19] Zhang N., Liu X., Jin X., Li C., Wu X., Yang S., Ning J., Yanne P. (2017). Determination of total iron-reactive phenolics, anthocyanins and tannins in wine grapes of skins and seeds based on near-infrared hyperspectral imaging. Food Chem..

[20] Cozzolino D., Kwiatkowski M.J., Parker M., Cynkar W.U., Dambergs R.G., Gishen M., Herderich M.J. (2004). Prediction of phenolic compounds in red wine fermentations by visible and near infrared spectroscopy. Anal. Chim. Acta.

[21] Slinkard K., Singleton V.L. (1977). Total Phenol Analysis: Automation and Comparison with Manual Methods. Am. J. Enol. Vitic..

[22] Ivanova V., Stefova M.T., Chinnici F. (2010). Determination of the polyphenol contents in Macedonian grapes and wines by standardized spectrophotometric methods. J. Serbian Chem. Soc..

[23] Osborne B.G., Fearn T., Hindle P.T. (1993). Practical NIR Spectroscopy with Applications in Food and Beverage Analysis.

[24] Siesler H.W., Ozaky Y., Kawata S., Heise H.M. (2002). Near Infrared Spectroscopy: Principles, Instruments, Applications.

[25] Hernández-Hierro J.M., Nogales-Bueno J., Rodríguez-Pulido F.J., Heredia F.J. (2013). Feasibility study on the use of near-infrared hyperspectral imaging for the screening of anthocyanins in intact grapes during ripening. J. Agric. Food Chem..

[26] Bautista-Ortín A.B., Jiménez-Pascual E., Busse-Valverde N., López-Roca J.M., Ros-García J.M., Gómez-Plaza E. (2013). Effect of Wine Maceration Enzymes on the Extraction of Grape Seed Proanthocyanidins. Food Bioprocess Technol..

